# A Novel Polyacrylamide Composite Hydrogel Reinforced with Deep Eutectic Solvent-Pretreated *Paulownia* Cellulose/Nanocellulose: Preparation, Characterization and Properties

**DOI:** 10.3390/gels12050411

**Published:** 2026-05-08

**Authors:** Hanyin Li, Yi Meng, Luohui Wang, Yan Gao, Youming Dong, Liangdi Zhang, Fei Xiao, Hanmin Wang, Cheng Li

**Affiliations:** 1 College of Forestry, Henan Agricultural University, Zhengzhou 450046, China; lihanyin@henau.edu.cn (H.L.);; 2 College of Materials Science and Engineering, Nanjing Forestry University, Nanjing 210037, China; youming.dong@njfu.edu.cn; 3 Hunan Academy of Forestry Sciences, Changsha 410018, China; 4 State Key Laboratory of Bio-Based Fiber Materials, Tianjin Key Laboratory of Pulp and Paper, Tianjin University of Science and Technology, Tianjin 300457, China

**Keywords:** forest biomass, Paulownia tree, deep eutectic solvent, nano-cellulose, hydrogel

## Abstract

Biomass represents a vital and sustainable resource for developing renewable materials with the potential to replace petroleum-based chemicals. Paulownia wood has high cellulose content and a loose wood structure, giving it natural advantages as a biomass material. Therefore, in this study, Paulownia wood was selected as a lignocellulosic feedstock. An integrated pretreatment process combining a deep eutectic solvent (DES) with an organic solvent was employed to efficiently remove lignin and hemicellulose, yielding cellulose-enriched residues. Subsequently, high-intensity ultrasonication was applied to convert the residues into cellulose nanofibers and nanocrystals. Using the extracted cellulose and nanocellulose, a dual-crosslinked network composite hydrogel was fabricated. The structural, mechanical, thermal, swelling, and conductive properties of the hydrogel were systematically investigated. The results show that, compared with the blank group hydrogel, the addition of nanocellulose increased the maximum tensile strength and tensile strain of the composite hydrogel by approximately 113% and 81%, respectively; meanwhile, the compressive strengths of the nanocellulose-based hydrogels (0.04575–0.09060 MPa) are higher than that of the blank group hydrogel (0.04235 MPa), confirming that the incorporation of nanocellulose significantly enhances the mechanical strength and elasticity of the hydrogel. The introduction of an AlCl_3_/ZnCl_2_ solvent system imparts appreciable electrical conductivity. Furthermore, the composite hydrogel maintains structural integrity after full swelling, indicating good dimensional stability and reusability. This work not only presents a green and efficient strategy for valorizing Paulownia biomass but also offers a novel design route for high-performance conductive hydrogel materials, highlighting their potential application in areas such as flexible electronics and energy storage.

**Abstract**

## 1. Introduction

The Paulownia tree, native to China, is a fast-growing broadleaf species known for its rapid growth, high adaptability, ease of harvesting, and excellent wood quality. With a cellulose content ranging from 60% to 70%, Paulownia wood serves as a rich source of lignocellulosic biomass [1,2]. Its porous structure and loose texture make it highly suitable for pretreatment, offering a natural advantage for biomass applications. As a model fast-growing species, Paulownia represents a promising and sustainable feedstock for biorefineries, aligning with global efforts towards a circular bioeconomy. By applying efficient pretreatment techniques, Paulownia wood can be effectively deconstructed to separate its main components and maximize cellulose utilization. This process enables the production of high-value-added products, significantly enhancing the economic potential of Paulownia while providing new strategies and technological support for the advanced utilization of lignocellulosic biomass [3]. The successful conversion of this underutilized resource into advanced materials could mitigate reliance on nonrenewable resources and offer environmental benefits.

Deep eutectic solvent (DES) is a transparent solvent formed by mixing specific ratios of hydrogen bond donors and acceptors under heating conditions [4]. Its physicochemical properties resemble those of traditional ionic liquids, including low vapor pressure, low melting point, high thermal stability, and strong polarity [5]. Moreover, DES is simple to synthesize and cost-effective to produce, making it an attractive alternative for biomass processing. The tunability of DES components allows for the design of solvents with specific properties tailored to different biomass feedstocks and target components, offering a versatile platform for green chemistry applications. In recent years, DES has been widely studied for its role in separating lignin, cellulose, and hemicellulose from lignocellulosic biomass [6,7]. By disrupting hydrogen bonds between lignin and carbohydrates, it effectively breaks down the rigid biomass structure, facilitating lignin removal and hemicellulose hydrolysis. This process yields cellulose-rich residues suitable for producing cellulose nanofibers. Compared to conventional methods, DES-based nanocellulose production offers advantages such as enhanced structural stability, environmental sustainability, and improved process efficiency [8,9]. The mild yet effective solvation power of DESs can preserve the native structure of cellulose to a greater extent compared to harsh chemical treatments, leading to nanocellulose with superior mechanical properties. Among various DES formulations, acidic DES is the most widely used and effective for biomass pretreatment. It effectively dissolves hemicellulose, degrades lignin or its complex linkages, and swells cellulose to further break down its fiber structure [10,11]. Oxalic acid, a naturally occurring and easily recoverable carboxylic acid [12], plays a key role in oxalic acid-based DES, effectively separating biomass components [13]. The use of oxalic acid, a dicarboxylic acid, is particularly advantageous, as it can act both as a hydrogen bond donor in the DES formation and as a mild acid catalyst for hydrolytic cleavage of glycosidic and ester bonds within the biomass matrix [14]. Oxalic acid-based DESs, such as choline chloride–oxalic acid (ChCl-OA), can also serve as hydrolysis media to depolymerize the amorphous regions of cellulose and release cellulose nanocrystals after mechanical processing. In this process, the unreacted free carboxyl groups in cellulose can enhance the dispersion of nanocellulose. This partial surface oxidation can facilitate subsequent chemical modifications or improve interfacial adhesion within composite materials. Acidic DES not only enables efficient cellulose conversion into high-value nanocellulose but also participates in cellulose surface derivatization and functionalization, expanding its application potential. Given its effectiveness in biomass pretreatment and nanocellulose production, DES holds significant promise for advancing lignocellulosic biomass utilization [15,16].

Hydrogels are condensed materials with a three-dimensional network structure capable of absorbing large amounts of water without dissolving. They are widely used in fields such as medicine, pharmaceuticals, and agriculture [17,18,19]. Cellulose-based hydrogels, including those derived from nanoscale cellulose, have shown significant potential in industries closely linked to daily life, such as food, healthcare, and wastewater treatment [20,21]. Their three-dimensional porous network, formed by water-absorbing functional groups, provides excellent biodegradability, high water absorption and swelling capacity, structural stability, and transparency. The integration of nanocellulose into hydrogel networks is a particularly promising strategy, as it combines the excellent water retention and softness of hydrogels with the high strength, stiffness, and large surface area of nanocellulose, resulting in mechanically reinforced and multifunctional nanocomposites [22]. These characteristics make cellulose-based hydrogels promising biodegradable materials with diverse applications in industrial products, agriculture, wastewater treatment, and biomedicine [23,24,25,26,27]. However, developing hydrogels with a balanced set of properties, such as high mechanical strength, excellent swelling capacity, and additional functionalities like electrical conductivity, remains a challenge, driving research into novel composite designs and crosslinking strategies.

In this study, cellulose and nanocellulose derived from DES-pretreated paulownia wood powder were incorporated into hydrogels to enhance structural stability. The AlCl_3_/ZnCl_2_ solvent system was introduced to improve the electrical conductivity of the hydrogel material [28]. This inorganic salt system, functioning as a Lewis acidic medium, not only facilitates the dissolution and dispersion of cellulose but also introduces mobile ions (Al^3+^, Zn^2+^, Cl^−^) into the hydrogel matrix, thereby imparting ionic conductivity, which is a property desirable for applications in sensors, actuators, and flexible electronics. Additionally, hydrogen bonding between cellulose and acrylamide was utilized, with ammonium sulfate as the initiator and *N*,*N*’-methylenebisacrylamide as the crosslinking agent, forming a dual-crosslinked network cellulose composite hydrogel. This hybrid network architecture, comprising both physical crosslinks (hydrogen bonds, chain entanglements, and nanocellulose reinforcement) and chemical crosslinks (covalent bonds from MBA), is designed to synergistically enhance the mechanical robustness, elasticity, and resilience of the hydrogel. The structure and mechanical properties of the composite hydrogel were systematically analyzed, providing new insights into the development of composite hydrogels. Furthermore, key properties, including swelling behavior, thermal stability, and electrical conductivity, were thoroughly investigated to establish comprehensive structure-property relationships. This work aims to demonstrate a sustainable and integrated approach for valorizing Paulownia biomass into high-performance, multifunctional hydrogel materials, thereby contributing to the development of advanced bio-based materials for emerging technologies.

## 2. Results and Discussion

### 2.1. Designation and Composition of the Hydrogels

To clarify the design and composition of the tested samples, the detailed designations and corresponding components of all hydrogel samples are summarized in Table 1. Briefly, the hydrogel samples included blank APM hydrogel (without cellulose/nanocellulose), cellulose-incorporated hydrogels (OC_1_/APM, OC_2_/APM, OC_3_/APM, OC_4_/APM, SU_1_/APM, SU_2_/APM, SUC_1_/APM, SUC_2_/APM) and nanocellulose-incorporated hydrogels (OC_1_-CNF/APM, OC_2_-CNF/APM, OC_3_-CNF/APM, OC_4_-CNF/APM), where the cellulose and nanocellulose were prepared under different DES pretreatment conditions.

### 2.2. Structure of Composite Hydrogels

**Figure 1:**
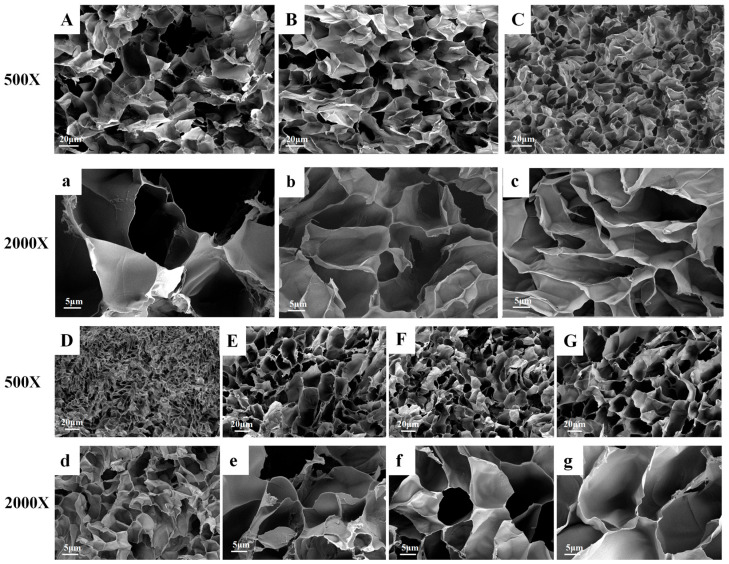
SEM images of APM, cellulose and nanocellulose composite hydrogels: APM (**a**,**A**), OC_2_-CNF/APM (**b**,**B**), OC_4_-CNF/APM (**c**,**C**), OC_2_/APM (**d**,**D**), OC_4_/APM (**e**,**E**), SU_2_/APM (**f**,**F**), and SUC_2_/APM (**g**,**G**).

Figure 1 shows the SEM images of APM and cellulose/nanocellulose hydrogels, revealing their internal microstructure. The hydrogel exhibited a characteristic honeycomb-like porous network, typical of hydrogel materials. The APM hydrogel, without cellulose or nanocellulose, had a dense yet porous network structure. With the inclusion of cellulose and nanocellulose, the hydrogel retained a well-defined three-dimensional network, facilitating the free diffusion of water molecules. The incorporation of cellulose and nanocellulose resulted in a denser pore structure due to enhanced crosslinking interactions, leading to a more compact and orderly network [29]. This increased crosslinking density not only reinforces the hydrogel structure but also affects its porosity, potentially influencing its swelling behavior and mechanical properties.

### 2.3. Mechanical Properties Analysis of Composite Hydrogels

**Figure 2:**
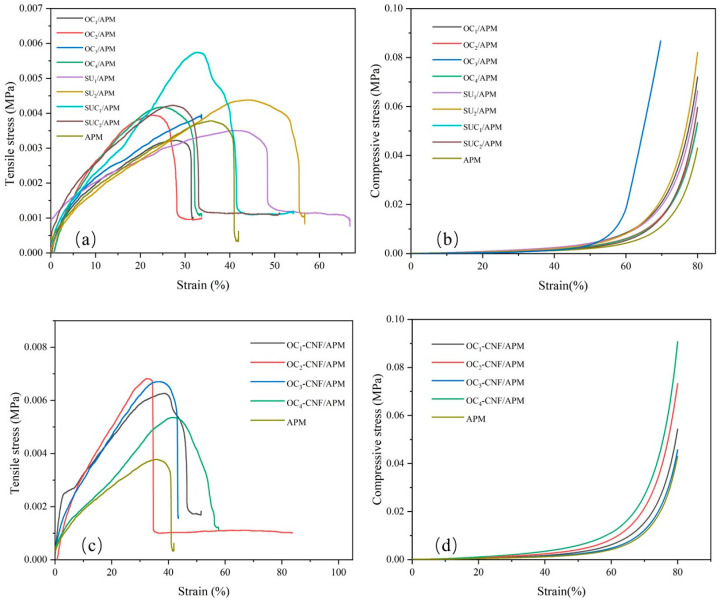
Tensile stress–strain curves (**a**,**c**) and compressive stress–strain curves (**b**,**d**) of cellulose and nanocellulose composite hydrogels.

The mechanical properties of the hydrogel are influenced by its chemical composition, energy dissipation mechanisms, and network topology. Figure 2 illustrates the tensile and compressive stress–strain curves of various cellulose and nanocellulose composite hydrogels. Figure 2a,b present the tensile and compressive stress–strain curves for APM and eight cellulose-derived composite hydrogels, respectively. Figure 2c presents the tensile stress–strain curves for APM and four cellulose nanofiber-based composite hydrogels, with Figure 2d depicting their compressive stress–strain behavior. Tensile strength and elongation at the break are key indicators of mechanical performance [30]. As shown in Figure 2a, the APM hydrogel exhibited a tensile strength of 0.0032 MPa with a tensile strain of 31%, while the eight cellulose-based hydrogels achieved a maximum tensile strength of 0.0057 MPa and a tensile strain of up to 55%. This indicates that cellulose addition significantly enhanced the tensile properties of the hydrogel, with hydrogels prepared using the amidosulfonic acid system exhibiting superior tensile strength and tensile strain compared to those using the oxalic acid system. In Figure 2c, the highest tensile strength among cellulose nanofiber composite hydrogels reached 0.0068 MPa, with tensile strength ranging from 0.0053 MPa to 0.0068 MPa and tensile strains between 24% and 56%. In contrast, cellulose-based hydrogels exhibited tensile strengths between 0.0039 MPa and 0.0043 MPa, with tensile strains of 27% to 34%. These results demonstrate that the incorporation of nanocellulose enhances hydrogen bonding and physical entanglement, thereby elevating network crosslinking density and structural flexibility, which leads to significantly improved mechanical properties relative to cellulose-based hydrogels [31,32].

Compression performance is a key property of hydrogels. Figure 2b shows the compressive strain-stress curves of cellulose-based composite hydrogels prepared using DES. At a strain of 80.15%, the APM hydrogel exhibited a compressive strength of 0.04235 MPa, indicating weak elasticity and poor compressive recovery. In contrast, at a strain of 70.97%, the compressive strength of cellulose-containing hydrogels achieved a compressive strength of up to 0.08670 MPa, demonstrating a significant improvement in elasticity. Figure 2d presents the compressive strain-stress curves of nanocellulose composite hydrogels. Under a strain of 80.08%, these hydrogels reached a maximum compressive strength of 0.09060 MPa, further enhancing elasticity with nanocellulose incorporation. The compressive strength of the nanocellulose-based hydrogels ranged from 0.04575 MPa to 0.09060 MPa, while that of the cellulose-based hydrogels ranged from 0.05339 MPa to 0.08670 MPa. Overall, nanocellulose-based hydrogels exhibited higher compressive strength than their cellulose counterparts, likely due to increased crosslinking density, which enhances compression performance. These findings suggest that nanocellulose significantly improves the mechanical properties of hydrogels.

### 2.4. Fourier Infrared Spectrum Analysis of Composite Hydrogels

**Figure 3:**
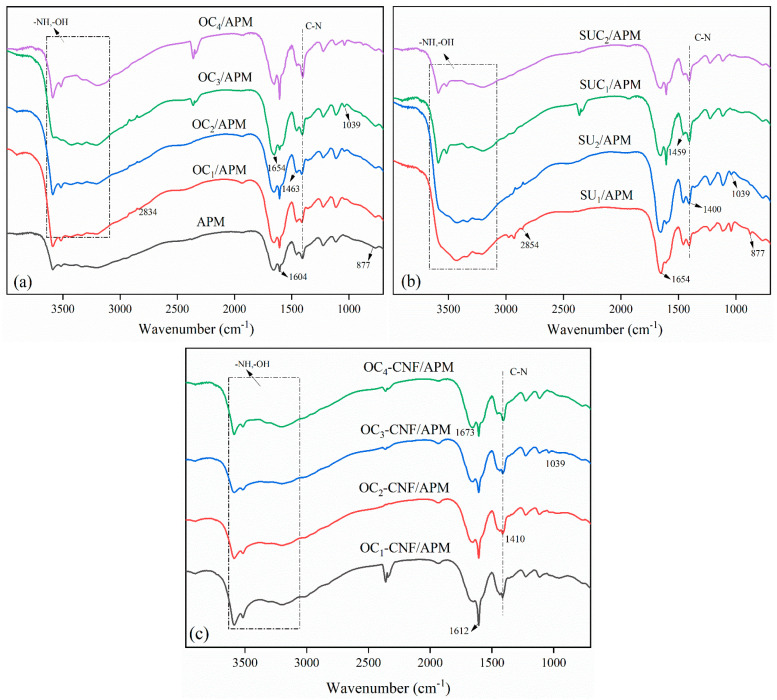
Infrared spectra of cellulose (**a**,**b**) and nanocellulose (**c**) composite hydrogels.

Figure 3 displays the infrared spectra of hydrogels prepared under different conditions. The stretching vibration of the N-H functional group appeared in the 3300–3500 cm^−1^ range [33], suggesting an interaction between the N-H bond in acrylamide and the O-H bond in cellulose, forming a broad peak. This confirms the successful incorporation of acrylamide into the hydrogel. The absorption peak at 1654 cm^−1^ corresponds to the C=O stretching vibration in acrylamide [34,35], further validating its presence. Characteristic peaks of cellulose hydrogels were observed at 1039 and 1421 cm^−1^, while a distinct C=C peak from acrylamide and *N*,*N*’-methylenebisacrylamide (MBA) appeared around 1604 cm^−1^ [36,37,38]. These findings collectively confirm the successful introduction of acrylamide and its interaction with cellulose.

### 2.5. Thermal Stability Analysis of Composite Hydrogels

**Figure 4:**
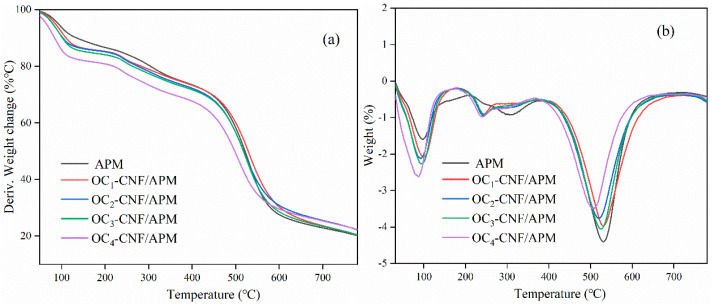
TGA (**a**) and DTG (**b**) curves of nanocellulose composite hydrogels. The OC_4_-CNF/APM sample exhibited slightly lower peak temperatures, possibly due to experimental conditions; however, the comparative trend is reliable.

Thermogravimetric analysis (TGA) is a crucial indicator of the thermal stability of cellulose materials. Figure 4 presents the TGA curve and the derivative thermogravimetric (DTG) curve of the nanocellulose composite hydrogel. The TGA results showed a gradual weight loss of approximately 15% to 30% between 50 °C and 220 °C, corresponding to water evaporation [18]. As the temperature increased, the sample exhibited different pyrolysis behaviors. The thermal degradation of the nanocellulose composite hydrogel began earlier, occurring between 220 °C and 400 °C. The DTG curve indicates that the peak of the maximum pyrolysis rate for the nanocellulose composite hydrogel occurred at 505 °C. Compared to APM, the composite hydrogel had a thermal degradation temperature of at least 20 °C lower, suggesting that the addition of nanocellulose slightly reduces thermal stability. This may be due to the shorter molecular chains of nanocellulose, making it more susceptible to thermal decomposition [39,40]. These findings further confirm the successful incorporation of nanocellulose into the hydrogel.

### 2.6. Swelling Performance Analysis of Composite Hydrogels

**Figure 5:**
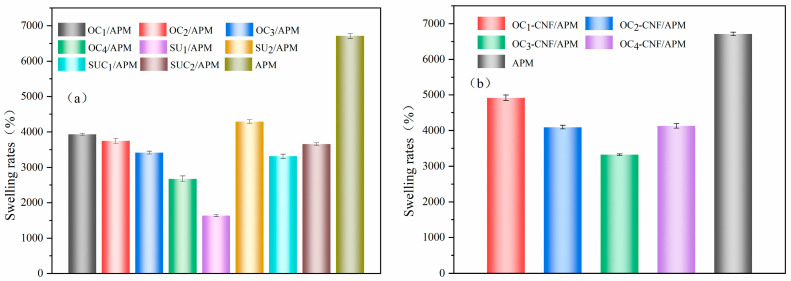
Swelling rates of cellulose (**a**) and nanocellulose (**b**) composite hydrogels.

The strong water absorption capacity of hydrogels stems from hydrophilic functional groups on the polymer backbone, while their resistance to dissolution results from crosslinking interactions between network chains. The swelling behavior of a hydrogel is typically expressed as the ratio of maximum water absorption (saturation) to the weight increase in the freeze-dried gel, with the swelling ratio serving as a direct measure of water absorption performance [41]. Figure 5 illustrates the swelling ratio variation with time for different hydrogels. While all samples demonstrated good water absorption, the swelling ratios of cellulose/nanocellulose composite hydrogels were lower than that of APM. This is because cellulose/nanocellulose acts as physical crosslinking points within the hydrogel. Once a certain level of swelling is reached, these crosslinking points restrict further water penetration, thereby reducing the swelling capacity [42]. However, the cellulose composite hydrogel maintained its shape stability after full swelling, making it advantageous for reuse.

### 2.7. Analysis of the Conductivity of Composite Hydrogels

**Figure 6:**
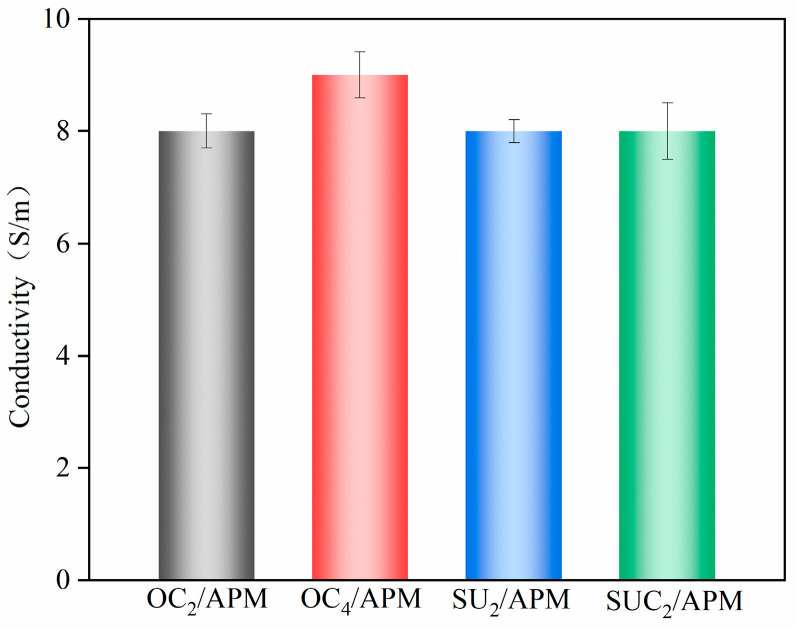
Conductivity of cellulose composite hydrogels.

A key challenge in cellulose-based hydrogels is the dense network structure formed by abundant hydroxyl groups, which hinders ion migration. Introducing an acrylamide network creates a porous structure within the hydrogel, improving ion migration. The conductivity results of four cellulose composite hydrogels are shown in Figure 6. The conductivity of OC_2_/APM, SU_2_/APM, and SUC_2_/APM hydrogels was 8 K/s, while OC_4_/APM reached 9 K/s, demonstrating good overall conductivity. This can be attributed to the hydrogel’s relatively loose network with larger pore sizes, which facilitates ion flow with minimal resistance [43]. Additionally, the use of the AlCl_3_/ZnCl_2_ solvent system, combined with the hydrogel’s porous structure, contributed to its excellent conductivity.

## 3. Conclusions

Using the AlCl_3_/ZnCl_2_ system, cellulose derived from paulownia wood powder pretreated with DES under different conditions, along with nanocellulose produced through high-intensity ultrasonication, was used to develop conductive cellulose and nanocellulose hydrogels. The microstructure, mechanical properties, chemical structure, swelling behavior, and conductivity of these composite hydrogels were investigated, offering a new approach to designing high-performance conductive gel materials. The results confirm the successful incorporation of acrylamide into the hydrogel, where it interacts with cellulose. Both cellulose and nanocellulose were effectively integrated into the hydrogel matrix. The incorporation of nanocellulose effectively improved the mechanical properties of the hydrogel as compared with the pure APM hydrogel. The maximum tensile strength and tensile strain of the composite hydrogel were increased by approximately 113% and 81%, respectively, and the compressive strength was enhanced from 0.04235 MPa to 0.04575–0.09060 MPa. These quantitative results confirm that nanocellulose reinforcement significantly optimizes the mechanical performance of hydrogels, providing a feasible strategy for the preparation of high-performance cellulose-based hydrogel materials. The cellulose composite hydrogels maintained structural stability after full swelling, demonstrating good swelling performance and reusability. Their dual-crosslinked network structure contributed to a well-developed pore architecture, while the AlCl_3_/ZnCl_2_ solvent system imparted excellent conductivity. This study provides an initial exploration of Paulownia cellulose in hydrogel applications. However, further improvements in the hydrogel performance are needed. This work has certain limitations: the long-term biodegradability of the hydrogel affected by AlCl_3_/ZnCl_2_ and the influence of ionic salt concentration on balancing electrical performance and mechanical robustness have not been systematically investigated. Additionally, given its unique physicochemical properties and microstructure, the material’s potential application in supercapacitors warrants further investigation.

## 4. Materials and Methods

### 4.1. Experimental Materials

Choline chloride was obtained from Shanghai McLin Biotechnology Co., Ltd. (Shanghai, China); oxalic acid dihydrate and aluminum chloride (AlCl_3_) from Shanghai Aladdin Biochemical Technology Co., Ltd. (Shanghai, China); and anhydrous ethanol from Tianjin Fuyu Fine Chemical Co., Ltd. (Tianjin, China). Amidosulfonic acid, urea, zinc chloride (ZnCl_2_), *N*,*N*’-methylenebisacrylamide (MBA), ammonium persulfate (APS), and acrylamide (AM) were all supplied by Shanghai McLin Biotechnology Co., Ltd.

### 4.2. Experimental Method

#### 4.2.1. Treatment of Paulownia Wood Powder

The trunks of 6-year-old Paulownia 9501 (Paulownia tomentosa × Paulownia fortunei 9501) trees, sourced from the Teaching and Experimental Base of Henan Agricultural University, were used as raw material. After debarking, drying, and grinding, Paulownia wood powder (40–60 mesh) was obtained. The powder was then subjected to continuous extraction using a Soxhlet extractor with a toluene–ethanol mixture (2:1, *v*/*v*) for 6–8 h. After complete solvent evaporation, the extracted Paulownia wood powder was dried in an oven at 60 °C.

#### 4.2.2. Extraction of Cellulose and Nanocellulose from Paulownia Wood Powder

In this experiment, three types of deep eutectic solvents (DES) were prepared: choline chloride–oxalic acid, amidosulfonic acid–urea, and choline chloride–amidosulfonic acid–urea, with molar ratios of 1:1, 1:2, and 1:2:1, respectively. The mixtures were stirred thoroughly in an 80 °C oil bath until a uniform transparent solution was obtained. Paulownia wood powder was then mixed with each DES type (choline chloride–oxalic acid, amidosulfonic acid–urea, and choline chloride–amidosulfonic acid–urea) at a solid-to-liquid ratio of 1:50 (g/g). The reaction was carried out under magnetic stirring at 500 rpm. The process was conducted at different temperatures (100 °C and 120 °C) and reaction times (3 and 6 h). After completion and cooling, solid–liquid separation was performed via vacuum filtration to obtain cellulose-rich residues. The reaction conditions for choline chloride–oxalic acid were labeled as OC_1_ (100 °C, 3 h), OC_2_ (100 °C, 6 h), OC_3_ (120 °C, 3 h), and OC_4_ (120 °C, 6 h); for amidosulfonic acid–urea, the two conditions were labeled as SU_1_ (100 °C, 3 h) and SU_2_ (100 °C, 6 h); and for choline chloride–amidosulfonic acid–urea, the two conditions were labeled as SUC_1_ (100 °C, 3 h) and SUC_2_ (100 °C, 6 h).

The cellulose residue pretreated with choline chloride–oxalic acid was ground and then mixed with deionized water to form a slurry with a solid content of 1 wt%. The suspension was then ultrasonically treated using a sonicator equipped with a 25 mm cylindrical titanium alloy probe. The process was conducted in an ice bath. The ultrasonic treatment was performed at a frequency of 20 kHz with an output power of 800 W, using a pulse cycle of 2 s on and 2 s off for 25 min. This process yielded a solution containing cellulose nanofibers. The resulting nanocellulose samples were labeled as OC_1_-CNF, OC_2_-CNF, OC_3_-CNF, and OC_4_-CNF.

#### 4.2.3. Preparation of Cellulose and Nanocellulose Composite Hydrogels

In total, 4.83 g of AlCl_3_ and 24.53 g of ZnCl_2_ were weighed according to their molar ratios and mixed with a crosslinking agent (MBA, 0.04 g), an initiator (APS, 0.04 g), and deionized water [44]. The mixture was stirred using a magnetic stirrer in a beaker to obtain a homogeneous metal salt solution. The extracted residual cellulose and nanocellulose were then added to the solution respectively (cellulose and acrylamide were pre-weighed at a mass ratio of 1:7), and stirred in a sealed state at room temperature until fully dissolved. Once a uniform cellulose solution was obtained, 2.1 g of acrylamide was added with continuous magnetic stirring for 30 min until the monomer was completely dissolved. The mixture was then heated to 80 °C until gelation occurred, forming a cellulose/acrylamide composite hydrogel [45]. The hydrogel without cellulose was labeled as APM. Hydrogels incorporating cellulose were labeled based on the type of cellulose used: OC_1_/APM, OC_2_/APM, OC_3_/APM, OC_4_/APM, SU_1_/APM, SU_2_/APM, SUC_1_/APM, and SUC_2_/APM. Hydrogels incorporating nanocellulose were labeled as OC_1_-CNF/APM, OC_2_-CNF/APM, OC_3_-CNF/APM, and OC_4_-CNF/APM, according to the type of nanocellulose used.

#### 4.2.4. Morphology and Structure Characterization of Composite Hydrogels

The morphology of the samples was observed and collected using a scanning electron microscope (Sigma 300, ZEISS, Jena, Germany). The hydrogel samples were fully solvent-exchanged with deionized water, then rapidly frozen and fractured in liquid nitrogen, followed by vacuum freeze-drying for 24 h to obtain test specimens. Before image acquisition, all specimens were mounted on the SEM stage with conductive adhesive, and their surfaces were coated with a gold layer via a sputtering coater. An accelerating voltage of 5 kV was applied during imaging.

#### 4.2.5. Determination of Mechanical Properties of Composite Hydrogels

The hydrogel samples were molded into cylindrical specimens with a diameter of 25 mm and a height of 10 mm using a polytetrafluoroethylene (PTFE) mold. The compression properties of the gel were tested using an electronic universal testing machine (RGM-6300, INSTRON, Norwood, MA, USA, 1 kN) at a test speed of 5 mm/min. For tensile testing, the hydrogels were cut into rectangular strips (gauge length 25 mm, width 10 mm, thickness 2 mm). The specimens were clamped with serrated fixtures equipped with soft rubber pads to avoid slippage and local damage during the stretching process, and their tensile stress–strain curves were measured using the electronic universal testing machine at a speed of 2 mm/min. All stress and strain data presented are true stress and strain. Each hydrogel sample was tested with three parallel replicates, and the average value was used for the final result.

#### 4.2.6. Determination of the Fourier Infrared Spectrum of Composite Hydrogels

The chemical structure of the composite hydrogels was analyzed by Fourier transform infrared (FT-IR) spectroscopy. Before analysis, the freeze-dried hydrogel samples were pulverized into a fine powder using an agate mortar and pestle. Each powdered sample was then meticulously homogenized with anhydrous potassium bromide (KBr) at a mass ratio of approximately 1:100 (sample: KBr). The homogeneous mixture was subsequently compressed under vacuum into a transparent pellet using a hydraulic press. FT-IR spectra were acquired using a Nicolet iS10 spectrometer (Thermo Fisher Scientific, Waltham, MA, USA) equipped with a DTGS detector. Spectral data were collected in the mid-infrared region, from 600 to 4000 cm^−1^, with a spectral resolution of 4 cm^−1^. Each spectrum represents an average of 32 scans to ensure an adequate signal-to-noise ratio.

#### 4.2.7. Determination of Thermogravimetry of Composite Hydrogels

The thermal decomposition behavior of the composite hydrogels was evaluated by thermogravimetric analysis (TGA) using a simultaneous thermal analyzer (STA8000, PerkinElmer, Shelton, CT, USA). Before analysis, the samples were desiccated in an oven until a constant mass was achieved. The dried materials were then thoroughly pulverized using an agate mortar and pestle, and the resulting powder was passed through a 200-mesh sieve to ensure uniform particle size. Approximately 10 mg of each freeze-dried sample was loaded into a platinum crucible. Measurements were performed under a continuous nitrogen purge (20 mL min^−1^) to maintain an inert atmosphere and prevent oxidative degradation. The temperature was programmed to ramp from 30 °C to 850 °C at a constant heating rate of 10 °C min^−1^. The resulting thermogravimetric (TGA) and derivative thermogravimetric (DTG) curves were recorded and analyzed to assess the thermal stability and degradation profiles of the materials.

#### 4.2.8. Determination of Swelling Degree of Complex Hydrogel

The hydrogel samples were immersed in deionized water for 48 h to reach swelling equilibrium. Afterward, the surface water was carefully removed, and the wet weight of each hydrogel sample was recorded. The swollen hydrogels were then freeze-dried for 24 h, and their dry weight was measured. All measurements were carried out in triplicate for statistical validation and error analysis. The swelling ratio (SR) was calculated using the following formula:

SR (%) = (Ws − Wd) / Wd × 100%

where Wd represents the dry weight of the hydrogel, Ws represents the wet weight of the hydrogel, and SR is the swelling ratio. The final results were presented as the mean ± standard deviation.

#### 4.2.9. Determination of Conductivity of Composite Hydrogels

The hydrogel samples were molded into block shapes with dimensions of 20 × 20 × 5 mm. These block samples were tested using an AC four-probe resistivity tester (ST2242) to measure the resistance. All measurements were carried out in triplicate for statistical validation and error analysis. The conductivity (σ) of the hydrogel was calculated using the following formula:

σ = L / RS

where L is the thickness of the hydrogel sample (cm), R is the measured resistance (Ω), and S is the contact area between the hydrogel and the copper plates (cm^2^). The final results were presented as the mean ± standard deviation.

## Figures and Tables

**Table 1:** Sample codes and compositions of the tested hydrogels.

Sample Code	Sample Type	DES System for Cellulose/Nanocellulose Preparation	Reaction Temperature for Cellulose/Nanocellulose Preparation	Reaction Time for Cellulose/Nanocellulose Preparation
APM	Control hydrogel	–	–	–
OC_1_/APM	Cellulose-based hydrogel	Choline chloride–oxalic acid	100 °C	3 h
OC_2_/APM	Cellulose-based hydrogel	Choline chloride–oxalic acid	100 °C	6 h
OC_3_/APM	Cellulose-based hydrogel	Choline chloride–oxalic acid	120 °C	3 h
OC_4_/APM	Cellulose-based hydrogel	Choline chloride–oxalic acid	120 °C	6 h
SU_1_/APM	Cellulose-based hydrogel	Amidosulfonic acid–urea	100 °C	3 h
SU_2_/APM	Cellulose-based hydrogel	Amidosulfonic acid–urea	100 °C	6 h
SUC_1_/APM	Cellulose-based hydrogel	Choline chloride–amidosulfonic acid–urea	100 °C	3 h
SUC_2_/APM	Cellulose-based hydrogel	Choline chloride–amidosulfonic acid–urea	100 °C	6 h
OC_1_-CNF/APM	Nanocellulose-based hydrogel	Choline chloride–oxalic acid	100 °C (further nanofibrillated)	3 h
OC_2_-CNF/APM	Nanocellulose-based hydrogel	Choline chloride–oxalic acid	100 °C (further nanofibrillated)	6 h
OC_3_-CNF/APM	Nanocellulose-based hydrogel	Choline chloride–oxalic acid	120 °C (further nanofibrillated)	3 h
OC_4_-CNF/APM	Nanocellulose-based hydrogel	Choline chloride–oxalic acid	120 °C (further nanofibrillated)	6 h

## Data Availability

All data and materials are available on request from the corresponding author. The data are not publicly available due to ongoing research using part of the data.
